# Effect of the Ethyl Acetate Fraction of *Eugenia uniflora* on Proteins Global Expression during Morphogenesis in *Candida albicans*

**DOI:** 10.3389/fmicb.2017.01788

**Published:** 2017-09-26

**Authors:** Walicyranison P. Silva-Rocha, Matheus F. de Azevedo, Magda R. A. Ferreira, Julhiany de Fátima da Silva, Terezinha I. E. Svidzinski, Eveline P. Milan, Luiz A. L. Soares, Keyla B. F. Rocha, Adriana F. Uchôa, Maria J. S. Mendes-Giannini, Ana M. Fusco Almeida, Guilherme M. Chaves

**Affiliations:** ^1^Laboratório de Micologia Médica e Molecular, Departamento de Análises Clínicas e Toxicológicas, Universidade Federal do Rio Grande do Norte, Natal, Brazil; ^2^Departamento de Farmácia, Centro de Ciências da Saúde, Universidade Federal de Pernambuco, Recife, Brazil; ^3^Faculdade de Ciências Farmacêuticas, Universidade Estadual Paulista, São Paulo, Brazil; ^4^Departamento de Análise Clínicas, Centro de Ciências Biológicas, Universidade Estadual de Maringá, Maringá, Brazil; ^5^Departamento de Infectologia, Centro de Ciências da Saúde, Universidade Federal do Rio Grande do Norte, Natal, Brazil; ^6^Departamento de Patologia, Centro de Ciências da Saúde, Universidade Federal do Rio Grande do Norte, Natal, Brazil; ^7^Departamento de Biologia Celular e Genética, Centro de Biociências, Instituto de Medicina Tropical do RN, Universidade Federal do Rio Grande do Norte, Natal, Brazil

**Keywords:** *Candida albicans*, phagocytosis, morphogenesis, proteomics, *Eugenia uniflora*, murine model

## Abstract

*Candida albicans* is able to switch from yeast to hyphal growth and this is an essential step for tissue invasion and establishment of infection. Due to the limited drug arsenal used to treat fungal infections and the constant emergence of resistant strains, it is important to search for new therapeutic candidates. Therefore, this study aimed to investigate by proteomic analysis the role of a natural product (*Eugenia uniflora*) in impairing hypha formation in *C. albicans*. We also tested the potential action of *E. uniflora* to prevent and treat oral candidiasis induced in a murine model of oral infection and the ability of polymorphonuclear neutrophils to phagocytize *C. albicans* cells treated with the ethyl acetate fraction of the extract. We found that this fraction greatly reduced hypha formation after morphogenesis induction in the presence of serum. Besides, several proteins were differentially expressed in cells treated with the fraction. Surprisingly, the ethyl acetate fraction significantly reduced phagocytosis in *C. albicans* (Mean 120.36 ± 36.71 yeasts/100 PMNs vs. 44.68 ± 19.84 yeasts/100 PMNs). Oral candidiasis was attenuated when *C. albicans* cells were either pre-incubated in the presence of *E. uniflora* or when the fraction was applied to the surface of the oral cavity after infection. These results were consistent with the reduction in CFU counts (2.36 vs. 1.85 Log10 CFU/ml) and attenuation of tissue damage observed with histopathological analysis of animals belonging to treated group. We also observed shorter true hyphae by direct examination and histopathological analysis, when cells were treated with the referred natural product. The *E. uniflora* ethyl acetate fraction was non-toxic to human cells. *E. uniflora* may act on essential proteins mainly related to cellular structure, reducing the capacity of filamentation and attenuating infection in a murine model, without causing any toxic effect on human cells, suggesting that it may be a future therapeutic alternative for the treatment of *Candida* infections.

## Introduction

Although some *Candida* species, mainly *C. albicans* belong to the normal human microbiota, they may become pathogenic in some circumstances, depending on host immunological conditions associated with virulence factors expressed by these microorganisms (Chaves et al., 2012; Younes and Khalaf, 2013). *C. albicans* is associated with superficial and systemic infections, and may cause life threatening diseases including candidemia, whose mortality rate is about 40–60% (Nucci et al., 2013).

Although *Candida* spp. live as commensals in the oral cavity of 30–60% of healthy individuals, these yeasts are capable of causing infection in the presence of predisposing conditions of the host, such as some environmental changes, which include a salivary gland dysfunction affecting the quantity and quality of saliva (Patil et al., 2015), the use of dental prosthesis, mainly of acrylic material, the presence of nutritional deficiencies, high carbohydrate diets, systemic diseases that may lead to imunnossupression, the use of immunosuppressive agents and organ transplantation (Sherman et al., 2002; Colombo et al., 2007; Muzyka and Epifanio, 2013; Nucci et al., 2013).

Oral candidiasis is frequently associated with high morbidity due the aspect of chronic pain which occurs in most cases, causing a discomfort upon mastication, which considerably alters the nutrition of individuals, mainly in the elderly and immunocompromised patients (Sherman et al., 2002).

Several virulence factors are associated with *C. albicans* pathogenicity, which contribute to the establishment of infection, including the ability to adhere to epithelial cells, the secretion of hydrolytic enzymes, biofilm formation and the transition of yeast cells to filamentous forms, which is known as morphogenesis (Silva-Rocha et al., 2015).

Another important virulence factor of *C. albicans* is the ability to combat reactive oxygen species (ROS) generated inside mammalian phagocytic cells, specifically polymorphonuclear neutrophils (PMNs; Marcos et al., 2016). Therefore, phagocytosis is the first line of defense against *C. albicans* invasion, and is performed by innate immune cells such as macrophage and neutrophils in healthy individuals (Dantas Ada et al., 2015) Neutropenia is a major risk factor for the establishment of *Candida* infections (Becker et al., 2015).

*C. albicans* resistance to phagocytosis by PMNs is due to the arsenal of enzymes produced by this yeast such as catalase, superoxide dismutase, glutathione reductase, which degrade reactive oxygen species produced by the immune host cells (Chaves et al., 2007; Herrero et al., 2008; Chaves and da Silva, 2012) and the ability to switch from budding to filamentous cells, which may cause a PMN membrane rupture allowing *C. albicans* to escape from PMNs killing (Dantas Ada et al., 2015).

Several studies related to morphogenesis in *C. albicans* were developed using mutant strains, whose genes that encode proteins responsible for these metabolic pathways, are attenuated in host tissue damage and virulence (Gow et al., 2012; Tsang et al., 2012). Jayatilake et al. (2006) demonstrated that *C. albicans* Δ*cph1/cph1*, a mutant with reduced ability of hypha formation, showed weaker tissue invasion and damage in a reconstituted oral epithelium model of infection, when compared to the wild type strain.

In the formation of true hyphae, an apical growth occurs to form tubular structures in the presence of parallels walls (Carlisle and Kadosh, 2013). The structures of filamentation in *C. albicans* are produced after the expression of several genes related to morphogenesis as well as by other co-regulated genes associated with other virulence factors, such as adherence and biofilm formation (Samaranayake et al., 2013; Childers and Kadosh, 2015). Morphogenesis is essential for the process of tissue invasion and thus the establishment of infection (Samaranayake et al., 2013; Childers and Kadosh, 2015).

The recent increase of antifungal resistance and the reduced therapeutic arsenal of drugs available in the management of fungal infections are still aggregated with other relevant problem that is the toxicity present in some classical antifungal drugs, such as amphotericin B, which causes renal toxicity (Hamill, 2013). Therefore, the investigation of natural products and their derived extracts from various vegetal parts (leaves, fruit, roots) for the purpose of seeking new compounds with antimicrobial properties has steadily increased recently (Amorim et al., 2009).

*Eugenia uniflora*, a widely distributed plant in South America, especially in Brazil, Argentina, Uruguay and Paraguay, is a tropical plant belonging to the family *Myrtaceae* and is popularly known as “pitangueira” in Brazil (Amorim et al., 2009). This plant family has more than 500 species, where 400 are natives of Brazil (Schumacher et al., 2015). It includes vegetables that have phenolic compounds with antioxidant and some with hypoglycemic and antirheumatic action, also used for stomach ailments and as antihypertensive (Amorim et al., 2009; Silva-Rocha et al., 2015). This natural product has also antimicrobial activity. This property was observed with its essential oil against *Staphylococcus aureus, Listeria monocytogenes* and *Candida* spp. (Victoria et al., 2012). The antifungal activity of several *E. uniflora* leaf fraction extracts were also observed against *Trichophyton rubrum* and *T. mentagrophytes* (Biasi-Garbin et al., 2016) and *Candida* species (Ferreira et al., 2013). Therefore, we used the ethyl acetate (EAF) fraction of this natural product by obtaining a fraction enriched in polyphenols. According to Ramos et al. (2017), the fraction has a flavonoid content about 9 times higher than the crude extract and this enrichment showed better anti-*Candida* activity. We decided to investigate whether this fraction, in addition to inhibiting fungal growth, could interact directly with the expression of virulence factors, specifically morphogenesis in *C. albicans*.

By evaluating transcriptional and proteomic responses to carbon starvation in *Paracoccidioides*, Lima et al. (2014) found that 86% of the matches showed the same pattern of transcripts and protein levels. Therefore, although we recognize that transcriptomics is more sensitive and comprehensive to study expression patterns, and that sometimes transcripts and proteins do not follow the same trend of expression that could be explained, for example, by mRNA stabilization process or by active post-transcriptional and translational regulatory mechanisms, proteomics may be a suitable and alternative less costly technique to study differential expression in fungi including *C. albicans* (Martínez-Gomariz et al., 2009; Gil-Bona et al., 2015).

Therefore, this study aimed to investigate which proteins are differentially expressed during filamentation when morphogenesis in *C. albicans* is induced in the presence of serum, after cell growth in the presence of the *E. uniflora* ethyl EAF. We also evaluated the direct interaction of the natural product with PMNs phagocytosis and performed a murine model of oral candidiasis in the presence of the referred fraction.

## Materials and methods

### Obtaining an enriched fraction of *Eugenia uniflora* linn leaves

The crude extract of *E. uniflora* leaves was obtained at 10% (w/v) with acetone: water (7:3, v/v) by turbo extraction in four rounds of 5 min. The extract was filtered and concentrated by rotary evaporation at 40°C (RV10 Basic, IKA®). The residue was frozen (−80°C, 3 days) and then lyophilized (Model L101, Liotop®) to yield a Crude Extract (CE). Approximately 20 g of CE were reconstituted in water (200 mL) and this solution was fractionated six times with 20 mL hexane. The resulting aqueous fraction was partitioned twelve more times with 20 mL ethyl acetate. This ethyl acetate fraction (EAF) was concentrated, frozen and lyophilized. The EAF was standardized as 17.82% m/m (expressed in rutin) of total flavonoids by UV/VIS and 6.56 % m/m (expressed as myricitrin) by HPLC (Ramos et al., 2017).

### Selection of microorganism

In order to evaluate *C. albicans* resistance to phagocytosis by polimorphonuclears neutrophils, (PMNs), 48 clinical isolates obtained from renal transplant recipients with oral candidiasis (Project approved under 152/07 protocol) and both references strains, *C. albicans* ATCC90028 and *C. albicans* SC5314 were used. These strains were kept in the culture collection of the Medical and Molecular Mycology Laboratory, Department of Clinical and Toxicological Analyses, Federal University of Rio Grande do Norte.

For all the other experiments, we selected *C. albicans* strain 111R. We previously observed this is a highly filamentous strain after induction of morphogenesis in the presence of serum and other morphogenesis inducing media (Silva-Rocha et al., 2015).

The isolates were stored at −80°C in YPD liquid medium (dextrose 20 g/L, peptone 20 g/L, yeast extract 10 g/L) containing 20% (v/v) glycerol. The cryotubes of 2 mL of capacity (Cralplast; Cotia Sao Paulo, Brazil) were thawed on ice and 100 μL of cells suspension of each strain were added to 5 mL of YPD liquid medium and incubated in a shaker afterwards (Tecnal, TE-420, São Paulo, Brazil), at 35°C for 48 h for the reactivation and verification of viability. Subsequently, 100 μL of each cell suspension was inoculated on the surface of Sabouraud Dextrose Agar (SDA; Oxoid, UK) containing 300 μg/mL of chloramphenicol (Park–Davis), using a Drigalsky loop. The plates were incubated at 37°C for 48 h. Yeast colonies were plated on CHROMagar® *Candida* (CHROMagar Microbiology, Paris, France) to check for purity and screening for different color colonies. Species identification was based on the characteristics of the cells observed microscopically after cultivation on corn meal agar containing Tween 80, as well as classical methodology (Yarrow, 1998).

### NGY broth to standardize the inoculum

For all the virulence factors evaluated *in vitro*, all *C. albicans* strains (phagocytosis) and 111R only (further assays) were initially grown in NGY medium (Difco Neopeptone 1 g/L, Dextrose 4 g/L; Difco yeast extract 1 g/L) for 18–24 h in a rotatory shaker (Tecnal, TE-420, Sao Paulo, Brazil) at 30°C, 200 rpm. This culture medium produces an inoculums size of about 2 × 10^8^ cells/mL. Cultures were spectrophotometrically measured at a wavelength of 600 _nm_ ranging from 0.8 to 1.2 (Biochrom Libra S32). Subsequently, *C. albicans* cells were diluted to obtain the specific inoculum needed for each attribute of virulence evaluated *in vitro* (Chaves et al., 2007). For the experiments performed after growth in the presence of the EAF of *E. uniflora*, 1,000 μg/mL of the natural product was added to the NGY broth. The experiments were performed in a manner that both control (in the absence of the EAF fraction) and test (in the presence of the EAF) assays had the same number of *C. albicans* viable cells after CFU counting, after 48 h of incubation in SDA.

### *Candida albicans* killing by polymorphonuclear neutrophils

PMNs freshly isolated from blood samples of the same healthy volunteer on the day of the experiment (Fradin et al., 2005) were suspended in Eagle's minimal essential medium (Gibco) + 20 mM HEPES, pH 7.2, and standardized to 8 × 10^5^ PMN/mL. *C. albicans* strain 111R cells grown overnight in NGY broth in the presence and absence of the EAF of *E. uniflora* were washed and resuspended to contain 5 × 10^6^ yeasts/mL in HEPES-buffered Eagle's minimal essential medium containing one-tenth volume of fresh human plasma for opsonization purposes. Equal volumes (100 μl) of PMNs and yeast suspensions were mixed and incubated at 37°C for 3 h with rotation at 50 rpm (Tecnal, TE-420, Sao Paulo, Brazil). Phagocytosis of *C. albicans* was determined by counting the number of yeasts cells inside or attached to 100 PMNs with optical microscopy (CX21, Olympus, Japan; Chaves et al., 2012). The assay was performed in triplicate.

### Morphogenesis assay in *Candida albicans*

Morphogenesis was induced according to Chaves et al. (2007). *C. albicans* cells were grown overnight in NGY broth (30°C–200 rpm) in the presence and absence of the EAF of *E. uniflora*. *C. albicans* cells were diluted in saline solution and standardized to 1 × 10^6^ cells/ml.

In order to induce morphogenesis, *C. albicans* cells suspension was inoculated to 150 ml of 20% (v/v) Fetal Bovine Serum (FBS; Sigma-Aldrich; Missouri, Saint Louis, USA) in YPD broth in 200 ml of capacity flasks. The strain was incubated under mechanical rotation at 37°C, 200 rpm, for a period of 3 h for further protein extraction.

### Proteomics analysis of *Candida albicans* treated with the EAF of *Eugenia uniflora*

A concentration of 1 × 10^6^ cells/ml of *C. albicans* 111R cells was used. After morphogenesis induction, *C. albicans* cells were transferred to 15 mL conic tubes (Cralplast; Cotia Sao Paulo, Brazil), centrifuged for 20 min, 13,000 rpm, 4°C (280R, FANEN) and washed three times with ice cold ultrapure water. Total protein extraction was performed with *C. albicans* cell pellets. Therefore, 5 ml of Tris-HCl 10 mM (pH 8.8) buffer containing a protease inhibitor solution cocktail (Protease Inhibitor Cocktail for use with mammalian cell and tissue extracts, DMSO solution, Sigma-Aldrich; Missouri, Saint Louis, USA) was added to the yeast cells (995 μL Buffer, 5 μL of protease inhibitor) subsequently macerated with liquid nitrogen. The samples were centrifuged at 13,000 rpm, 4°C and the supernatant was collected. The total protein concentration of the supernatant was quantified by Bradford method (Bradford, 1976) using Bovine Serum Albumin (BSA; Sigma-Aldrich; Missouri, Saint Louis, USA) as a standard and the absorbance readings were performed at 595 wavelength by spectrophotometer.

Protein profiles were analyzed with Sodium Dodecyl Sulfate Polyacrylamide Gel Electrophoresis, SDS-PAGE, under reducing conditions using the discontinuous buffer system (Laemmli, 1970; Studier, 1973).

Total soluble proteins were separated and analyzed by two-dimensional electrophoresis (Görg et al., 2000). Firstly the proteins were submitted to Ettan IPGphor 3 (GE Healthcare) according to the following parameters: First gradient—500 V, second gradient—1,000 V, third gradient—8,000 V, fourth gradient—8,000 V, 20°C, 50 mA. The second dimension was carried out for the separation of proteins according to molecular mass, and held on 12.5% (w/v) polyacrylamide gel according to Laemmli (1970). Gels from tests and control experiments extracted proteins were stained with Coomassie Brilliant Blue G-250, according to Neuhoff et al. (1988). The gels were scanned (Labscan, HuntersLab). The analysis of data was performed with the ImageMaster 2D Platinum 6.0 GE software.

The spots resulting from gel electrophoresis were then removed and cut into segments of ~1 mm^3^. The fragments were subsequently destained with 400 μL of decolorizing solution containing acetonitrile (ACN) 50% (v/v) in ammonium bicarbonate 25 mM, stirred with a vortex for 10 min between each washing until the complete removal of the dye. Gel excised fragments were then completely dehydrated with 200 μL of 100% ACN and than dried in a concentrator (Vacfuge Plus™, Eppendorf). The fragments were subsequently rehydrated with 40 μL of Dithiothreitol (20 mM DTT in 50 mM ammonium bicarbonate) for 40 min at 56°C. After rehydration was carried out, alkylation with 40 μL of iodoacetamide solution (55 mM iodoacetamide in 50 mM ammonium bicarbonate) was performed. A second dehydration was performed with 200 μL of ACN (100%). Subsequently, 15 μL of trypsin solution in acetic acid (100 ng/μL) was added to the dried gel fragments which were then rehydrated in ice. A 40 μL volume of 25 mM ammonium bicarbonate was added and the fragments were incubated at 37°C for 14h.The reaction was stopped with 15 μL of blocking solution (ACN 50% (v/v) and formic acid 5% (v/v). Three elutions were carried (repated twice each time) and supernatants were collected after all elutions. The first elution was performed with 40 μL of methanol 60% (v/v) and formic acid 1% (v/v), and then the samples sonicated for 15 min at 40°C. For the second elution 40 μL of ACN 50% (v/v) and formic acid 1% (v/v) was added and then samples were sonicated for 15 min at 40°C. A volume of 40 μL of ACN 100% was added to dehydrate gel fragments, subsequently sonicated for 15 min. The eluted peptides were then subjected to concentration in a Concentrator (Vacfuge Plus™, Eppendorf) during 30 min. After concentration, samples were resuspended in 10 μL of trifluoroacetic acid (TFA) 0.1% (v/v) in 100% ACN and subjected to purification before mass spectrometry analysis.

Purification was performed using Reverse Phase-Pippete Zip-Tip (Millipore) following the manufacturer's instructions. Peptide samples were resuspended in 0.1% (w/v) TFA solution. The ZipTips were equilibrated with 10 μL of a solution containing ACN and 0.1% TFA and the solution discarded afterwards. This procedure was performed three times. A 10 μL volume of water and 0.1% TFA solution was gently homogenized and further discharged for three times. To bind the samples inside the pipette tips to columns, 10 μL of sample peptides were aspirated for 7 to 10 times to increase fixation to columns. After binding of the peptides to columns, two washing steps were performed. Firstly, 10 μL of a 5% (v/v) methanol and 0.1% TFA solution was added and the process repeated three times. The elution process was ended after gentle homogenization of peptides the addition of 5 μL of 0.1% TFA solution in 50% ACN and subsequently the samples were transferred to clean 2 mL microcentrifuge tubes (Salvato and Carvalho, 2010).

After trypsin digestion and purification, the peptides were then analyzed by mass spectrometry. The peptides were identified by mass spectrometry by MALDI-TOF/TOF equipment (TOF/TOF™ 5800 System, Sciex), the fragmentation spectra (MS/MS) containing the molecular weights of the peptides were evaluated using the MASCOT database (MatrixScience). Morphogenesis assays and proteomic analysis were perdormed in triplicate.

### Murine model of oral candidiasis

The evaluation of the effect of the EAF of *E. uniflora* on filamentation of *C. albicans* was performed *in vivo* according to Solis and Filler (2012). Twenty-four adult mice (*Mus musculus*) of 12 weeks old weighing 18–25 g were used. The animals were obtained from the Animal House of the Center of Health Sciences, Federal University of Rio Grande do Norte, kindly provided by Dr. Antonia Cláudia Jácome da Câmara. Water and food were provided *ad libitum* throughout the study period. The day before animal's experimentation, *C. albicans* 111R was grown overnigth in NGY broth in the absence (control experiment) and presence (test experiment) of 1,000 μg/mL of the EAF of *E. uniflora* at 30°C, 200 rpm for 18–24 h. The animals were weighed a day before inoculation to calculate the dose of the immunosuppressant (prednisolone, EMS; Hortolandia, Sao Paulo, Brazil) subcutaneously administered to each individual (100 mg/kg in 200 μL). Prednisolone was administered a day previously to infection and 3 days after infection. An inoculum of 1 × 10^6^ cells of *C. albicans* 111R strain was prepared in PBS buffer. The animals were intraperitoneally anesthetized with a mixture of ketamine (Francotar®, Virbac;110 mg/kg) and xylazine (Calmiun®, Agener União) (10 mg/kg). After anesthetizing the animals, a sterile swab was inserted in microcentrifuge tubes containg *C. albicans* inoculum, all the cells suspension (100 μL) was dropped and rubbed on the oral cavity of the animals during 60 s. CFU counts were performed before inoculation to verify if cells were viable.

The animals were divided in three different groups: Group I: Animals that were infected with *C. albicans* strain 111R grown in NGY broth the absence of *E. uniflora* EAF. Group II: Animals that were infected with *C. albicans* strain 111R grown in NGY broth in the presence of *E. uniflora* EAF and Group III: Animals that were infected with *C. albicans* strain 111R grown in NGY broth in the absence of *E. uniflora* EAF, where the fraction was administered to the animals tongue after infection.

The animals from group III were intraperitoneally anesthetized with a mixture of ketamine (110 mg/kg) and xylazine (10 mg/kg) 2 days after the infection was established, and were subsequently treated by admiministring 50 μL of the *E. uniflora* EAF (1,000 μg/mL) onto the dorsum of the tongue for 3 times in intervals of 10 min.

The animals were sacrificed 5 days after infection with excessive dose of ketamine (110 mg/kg) and xylazine (10 mg/kg). A sterile swab was rubbed on the oral cavity of animals and introduced into a microcentrifuge tube containing 1 ml of 0.9% (w/v) saline. The samples were diluted 1:10 and seeded on the surface of 90 × 120 mm Petri dishes containing Sabouraud dextrose agar with 300 μg/mL chloramphenicol and incubated at 37°C for 48 h to perform CFU counting.

The animals tongues were excised with the aid of a sterile scalpel and immediately fixed in formaldehyde 10% (v/v) in PBS for further histopathological analysis of the tissue. After fixation steps, the samples were dehydrated with graded ethanol and diaphanized xylene. The samples were embedded in paraffin and sliced in eight mm of thickness, for the manufacture of the slides and then stained with hematoxylin-eosin for subsequent evaluation step of the cellular infiltrate and PAS (Periodic Acid Schiff) to assess the fungal load in a light microscope. In order to evaluate tissue damage resulting from the infection, the formation of tissue ulcers, erosion process, inflammatory response, as well as the type of response and presence and fungal burden at the infection site were evaluated.

### Cell viability assay

Cell viability was studied in A549 cell lines at a concentration of 2.5 × 10^2^ cells per well. *E. uniflora* ethyl acetate fraction was tested in concentrations of 8,000, 4,000, 2,000, 1,000, 500, 250, 125, 62.5, 37, and 18 μg/mL. Cytotoxicity was assessed by the MTT assay (bromide (3- [4,5-dimethyl-thiazol-2-yl] -2,5-diphenyltetrazolium bromide) (Sigma-Aldrich; Missouri, Saint Louis, USA), used at a concentration of 5 mg/ml according with Mosmann (1983) with some modifications.

The starting solution with the EAF fraction of *E. uniflora* was subsequently serially diluted in the specific medium used for cell line in the previously prepared microplates containing the mammalian cells. The plates were incubated at 37°C, 5% CO_2_ under light for 24 h. The solution containing the fraction was removed and 10 μL of MTT solution was added to the wells, following another incubation step at 37°C for a period of 4 h, as previously described (Denizot and Lang, 1986; Gerlier and Thomasset, 1986; Ferrari et al., 1990). After the incubation time, the MTT solution was removed and added to 100 μL of isopropanol for diluting the precipitate. Viable cells changed their color from yellow to violet. Untreated cells constituted the positive control (viable cells), and cells treated with hydrogen peroxide (Sigma-Aldrich, St. Louis, MO, USA) constituted the negative control (dead cells). The spectrophotometric reading of the plates was performed in the microplate ELISA reader (BioRad model 3550) with a wavelength of 570 nm. The percentage of viable cells was calculated as follows:

$$\begin{array}{l} \text{Viable cells }\%=\text{average test X}100/\text{average of negative control} \end{array}$$

## Results

### *Candida albicans* killing by polymorphonuclear neutrophils

In a previous study performed by our group, we realized that the extract of *E. uniflora* was able to impair proper hypha formation in both solid and liquid media (YPD+20% serum, Spider and N-acetyl-D-glucosamine; Silva-Rocha et al., 2015). In addition, the typical oval morphological form of *C. albicans* blastoconidia was altered; showing invaginations of the cell wall and an increase in the size of citoplasmic vacuoles. We hypothesized that the extract was somehow acting on the cell wall, and then we investigated how *C. albicans* cells grown in the presence of *E. uniflora* would interact with phagocytic cells. Therefore, we performed phagocytosis assays with PMNs.

PMNs were co-incubated with *C. albicans* cells that were previously grown overnight in the presence and absence of the EAF of *E. uniflora*. In the absence of the ethyl acetate fraction, *C. albicans* strains had a broad variability of number of yeasts phagocytized by 100 PMNs, ranging from 11 ± 3.2 (Strain 111R) to 176 ± 11.6 and 176 ± 6.1 (Strains 44 and 10Li, respectively) yeasts/100 PMNs. The mean value of the number of *C. albicans* phagocytized by 100 PMNs for all the strains was 120.36 ± 36.71 yeasts/100 PMNs (Table 1).

**Table 1:** Phagocytosis by polymorphonuclear neutrophils of *Candida albicans* clinical isolates obtained from the oral cavity of kidney transplant recipients after 3 h of incubation in Eagle's minimal essential medium buffered with HEPES, pH 7.2 media at 37°C, 200 rpm.

**Strain**	No of *C. albicans* cells phagocitized by 100 PMNs^*^	**Strain**	No of *C. albicans* cells phagocitized by 100 PMNs^*^
ATCC90028	158 ± 17.5 vs. 17 ± 6.5^†^	Strain44	176 ± 11.6 vs. 63 ± 7.5^†^
SC5314	137 ± 20.6 vs. 16 ± 3.5^†^	Strain46	172 ± 20.1 vs. 91 ± 9.1^†^
Strain01	134 ± 15.3 vs. 26 ± 1.2^†^	Strain50	157 ± 36.6 vs. 52 ± 10^†^
Strain02	115 ± 14.6 vs. 14 ± 3.2^†^	Strain51	130 ± 19.5 vs. 74 ± 13.7^†^
Strain03	66 ± 7.9 vs. 20 ± 0.6^†^	Strain53	132 ± 15.9 vs. 49 ± 10.3^†^
Strain05	98 ± 6.7 vs. 48 ± 8.2^†^	Strain54	132 ± 9.2 vs. 46 ± 11.1^†^
Strain06	100 ± 12.5 vs. 23 ± 6.5^†^	Strain60	146 ± 9 vs. 57 ± 11.6^†^
Strain08	130 ± 19 vs. 50 ± 9^†^	Strain61	142 ± 9.2 vs. 56 ± 11.1^†^
Strain10	166 ± 8.4 vs. 35 ± 12.5^†^	Strain70	140 ± 12.7 vs. 61 ± 9.9^†^
Strain11	28 ± 3.5 vs. 14 ± 2.5	Strain72	136 ± 9 vs. 67 ± 11.6^†^
Strain12	150 ± 11.1 vs. 81 ± 15.4^†^	Strain82	130 ± 13.2 vs. 51 ± 9.9^†^
Strain13	126 ± 31.6 vs. 42 ± 4^†^	Strain85	144 ± 9.5 vs. 53 ± 6.1^†^
Strain17	98 ± 20.2 vs. 70 ± 4^†^	Strain107	156 ± 10.1 vs. 71 ± 9.9^†^
Strain20	86 ± 21.2 vs. 39 ± 17.2^†^	Strain06A	80 ± 17.4 vs. 37 ± 6.1^†^
Strain21	114 ± 21.5 vs. 37 ± 10.6^†^	Strain06L	75 ± 8.7 vs. 24 ± 1^†^
Strain23	101 ± 11.4 vs. 62 ± 4.6^†^	Strain10Li	176 ± 6.1 vs. 34 ± 2^†^
Strain24	132 ± 13.9 vs. 57 ± 8.5^†^	Strain10S	105 ± 14.7 vs. 41 ± 14.4^†^
Strain28	165 ± 9.5 vs. 76 ± 13.4^†^	Strain12A	59 ± 16.8 vs. 15 ± 4.2^†^
Strain30	119 ± 20 vs. 44 ± 17.6^†^	Strain12L	90 ± 13.2 vs. 28 ± 3.5^†^
Strain31	129 ± 15 vs. 56 ± 14.5^†^	Strain15A	116 ± 27.1 vs. 39 ± 7^†^
Strain32	123 ± 13.2 vs. 60 ± 6.1^†^	Strain15G	86 ± 21.9 vs. 34 ± 3.1^†^
Strain34	156 ± 16.5 vs. 67 ± 8.2^†^	Strain15L	76 ± 16.5 vs. 38 ± 4^†^
Strain37	124 ± 19.1 vs. 41 ± 17.6^†^	Strain18A	102 ± 11.2 vs. 10 ± 1^†^
Strain40	142 ± 15.9 vs. 59 ± 10.3^†^	Strain111L	80 ± 17.4 vs. 37 ± 6.1^†^
Strain41	172 ± 21.1 vs. 42 ± 14.6^†^	Strain111R	11 ± 3.2 vs. 10 ± 5.5

* *Presence vs. Absence of EAFof Eugenia uniflora extract*.

† *Statistically significant P < 0.05*.

**Figure 1:**
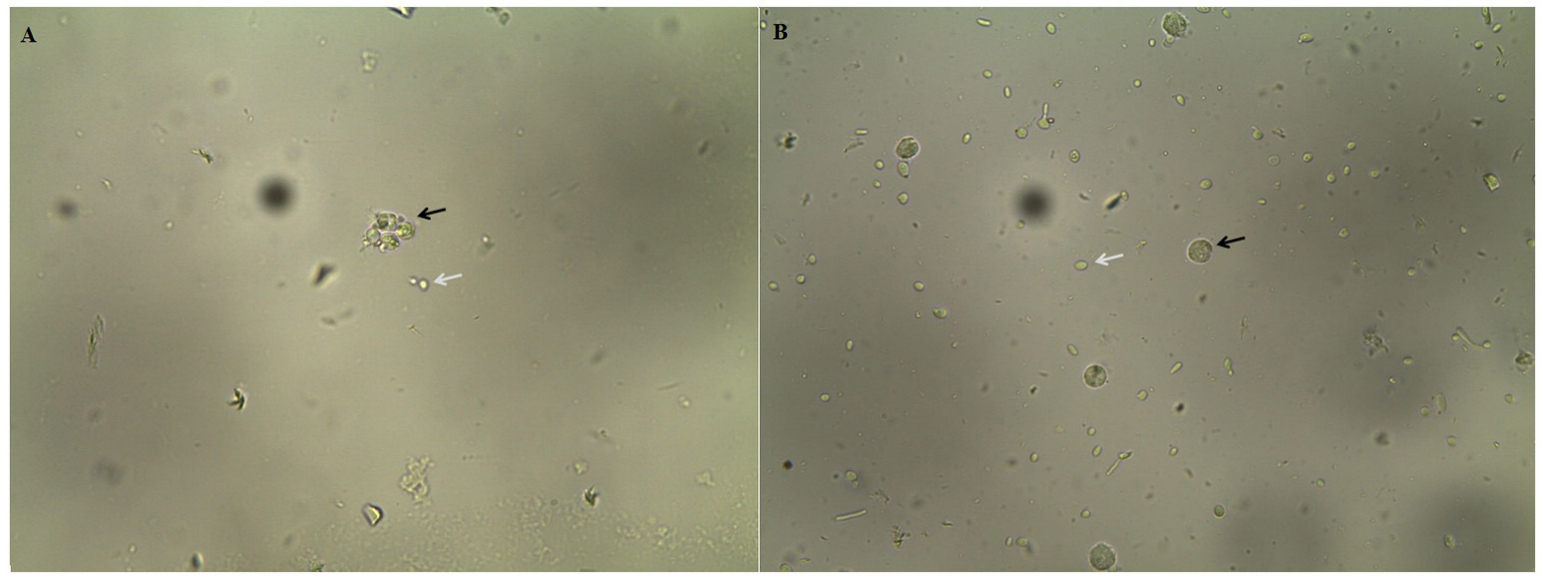
Phagocytosis of *Candida albicans* SC5314 cells by polymorphonuclear neutrophils after 3 h of incubation in Eagle's minimal essential medium buffered with HEPES, pH 7.2 media at 37°C, 200 rpm. **(A)** *C. albicans* cells grown overnight in NGY broth at 30°C, in the absence of *E. uniflora* extract (Black arrow: *C. albicans* inside PMNs. White arrow: Blastoconidia non-phagocytized). **(B)** *C. albicans* cells grown overnight in NGY broth containing *E. uniflora* EAF (1,000 μg/mL). Black arrow: PMN; White arrow, *C. albicans* blastoconidia.

When *C. albicans* cells were grown overnight in the presence of *E. uniflora* extract, a significant reduction in the number of cells phagocytized by the PMNs was observed (Figure 1B). The number of *C. albicans* phagocytized by 100 PMNs ranged from 10 ± 1 to 10 ± 5.5 yeasts/100 PMNs (Strain 18A and 111R) to 91 ± 9.1 yeasts/100 PMNs (Strain 46; Table 1). A comparison among the mean value of phagocytosis of the control group (absence of the EAF) with the test group shows a significant reduction in the number of cells phagocytized by PMNs (120.36 ± 36.71 vs. 44.68 ± 19.84 yeasts/100 PMNs; Table 1). Phagocytosis of *C. albicans* cells by PMNs and hypha formation may be observed when cells were cultivated in the absence of the EAF of *E. uniflora* (Figure 1A).

Surprisingly, when *C. albicans* cells were grown overnight in the presence of the *E. uniflora* EAF, a significant reduction in the number of cells phagocytized by the PMNs was observed (Figure 1B). The number of *C. albicans* phagocytized by 100 PMNs ranged from 10 ± 1 to 10 ± 5.5 yeasts/100 PMNs (Strain 18A and 111R) to 91 ± 9.1 yeasts/100 PMNs (Strain 46; Table 1). A comparison among the mean value of phagocytosis of the control group (absence of the fraction) with the test group shows a significant reduction in the number of cells phagocytized by PMNs (120.36 ± 36.71 vs. 44.68 ± 19.84 yeasts/100 PMNs; Table 1).

It is important to emphasize again that *C. albicans* 111R is a highly filamentous strain found in our previous publication (Silva-Rocha et al., 2015). Due to its highly filamentous phenotype, which can be easily observed even when this strain is not incubated in hypha inducing conditions; this strain was not promptly phagocytized. Therefore, we could not observe a significant reduction in the number of phagocytized cells when this strain was previously grown in the presence of the EAF, because this strain was already poorly phagocytized (*P* > 0.05). However, because this was the most filamentous strain found in our study and we would like to prove that this natural product was able to impair morphogenesis, we chose it to perform our proteomic assays.

### Global analysis of *Candida albicans* proteomics in morphogenesis induced with fetal bovine serum

Because we observed that *C. albicans* 111 R was the most filamentous strain, but also showed reduced ability to form filaments in the presence of inducing media after growth in the presence of the EAF, we selected this strain to analyze which proteins are differentially expressed in the of the referred fraction. The induction of filamentation was performed in YPD+20% serum at 37°C, for 3 h and proteins were analyzed by bidimensional electrophoresis. A total of 39 proteins, conserved in the three different 2D-gels (triplicate assays), were selected and then identified by MALDI-TOF mass spectrometry. Proteins identification and their biological functions are described in Table 2.

**Table 2:** Effect of EAF of *Eugenia uniflora* (1,000 μg/mL) on global protein profile of *Candida albicans* 111R strain after morphogenesis induction in fetal bovine serum after 3 h of incubation at 37°C, 200 rpm.

**Spot ID**	**Protein**	**Gene**	**Biologycal process**	**Accession number**	**Molecular weight**	**Isoelectric point**	**Extract effect**
1	Mitochondrial import inner membrane translocase subunit TIM16	PAM16	Mitochondrial biogenesis	Q59ZW9	13,306	9.99	Increase
2	Mediator of RNA polymerase subunit 7	MED7	Glycolytic pathway promoter	Q08278	25,585	6.95	Increase
4	Ucharacterized protein	IRC10	Unknown function	Q08118	67,689	9.74	–
5	F1F0-ATPase complex. F1 beta subunit	ATP2	ATP synthesis	Q59UR7	55,728	4.68	Increase
7	GTP-Binding Protein 1	GTP1	Protein syntesis	P32235	40,848	5.78	Decrease
8	Ucharacterized protein	SPAC24C9.04	Unknown function	O13964	13,789	6.16	–
10	Kinetochore protein Nuf-2	NUF2	Cell division	Q5A1Q5	56,648	4.25	Decrease
11	Peptidyl-prolyl cis-trans isomerase 9	CYP9	Protein syntesis	O74942	69,048	5.62	Decrease
12	Respiratory supercomplex factor 1. mitochondrial	RCF1	Mitochondrial biogenesis	Q59N74	15,632	9.64	Increase
13	Cohesin	PDS5	Cell division	Q04264	147,041	6.18	–
14	60S large subunit ribosomal protein L3	RPL3	Protein syntesis	Q59LS1	43,948	10.94	–
15	Spore Wall Protein 12	SWP12	Cell division	Q8SWP1	27,357	9.23	–
16	Mitochondrial outer membrane protein	CAGL0G03245g	Mitochondrial biogenesis	Q6FTE0	85,129	5.17	–
22	Syntaxin-binding protein 1	SEC1	Protein transport	Q5AH58	89,635	8.00	–
23	Enolase 1	ENO1	Glycolytic pathway—Growth	P30575	47,231	5.52	Increase
24	Fructose-bisphosphate aldolase	FBA1	Glycolytic pathway—Growth	Q9URB4	39,215	5.97	–
26	Polynucleotide 3′-phosphatase	TPP1	DNA repair	Q03796	27,383	8.95	–
27	Proteasome Subunit Alpha-2	PCA2	Protein Catalysis	Q8X077	26,998	5.72	–
28	54S ribosomal protein L4	MRPL4	Ribossomal structure	Q59RP7	36,392	5.71	–
29	26S Proteasome regulatory subunit N6	RPN6	Protein Catalysis	Q59TR9	49,384	6.04	–
30	Acyl-CoA-binding protein 2	ACB2	Protein transport	P61868	10,084	4.74	Decrease
31	Sterol 3-beta-glucosyltransferase	ATG26	Membrane synthesis	Q5A950	170,545	5.57	–
32	ATP-dependent RNA helicase DBP10	DBP10	Ribossomal structure	Q5ANB2	103,561	9.64	–
34	U6 snRNA-associated Sm-like protein LSm3	LSM3	RNA processing	P57743	10,030	4.29	–
35	Actin cytoskeleton-regulatory complex protein END3	END3	Endocytosis; Growth	Q5AJ82	44,983	4.64	–
36	Carboxypeptidase 2	MCPB	Extracellular metalloprotease	D4D4Z1	57,640	8.30	–
37	RNA exonuclease 3	REX3	RNA processing	Q5AL29	46,495	8.77	–
38	Meiotically up-regulated gene 97 protein	MUG97	Cell division	Q9Y800	39,385	8.61	Decrease
40	Nitrate reductase	YNR1	Nitrate assimilation	P49050	98,534	5.97	Increase
41	Stationary phase protein 4	SPG4	Stationary phase	B4UN34	10,753	6.91	Increase
42	Heat shock protein SSB1	SSB1	Protein biosynthesis. Stress response	P87222	66,479	5.01	Increase
43	Cytochrome c oxidase subunit 4. mitochondrial	COX4	ATP synthesis	P79010	18,162	8.78	Decrease
44	Endoplasmic reticulum transmembrane protein 2	YET2	Protein transport	Q04210	19,178	10.21	Decrease
45	Elongation factor 1-alpha 1	TEF1	Protein syntesis	P0CY35	50,012	9.47	–
46	Protein MTH1	MTH1	Glucose transport. signal transduction	P35198	49,060	9.49	Decrease
47	Nucleoside diphosphate kinase	NDK1	ATP synthesis. Nucleotide metabolism	Q9UR66	2,379	4.75	Decrease
49	ATP-dependent RNA helicase MRH4. mitochondrial	MRH4	Ribossomal structure	Q59S59	62,513	9.70	–
52	Protein PET191. mitochondrial	CAWG_02398	ATP synthesis	C4YPJ7	13,543	8.86	–
59	Prefoldin subunit 2	GIM4	Protein folding	P40005	14,331	6.62	Decrease

The proteins identified were classified within seven distinct categories based on their biological function, as follows: Energy (Pam16, Atp2, Rcf1, Cagl0g03245g, Cox4, Ndk1, and Cawg_02398), Protein Metabolism (Gtp1, Cyp9, Rpl3, Pca2, Mrpl4, Rpn6, Dbp10, Tef1, and Mrh4), Glucose Metabolism (Eno1, Fba1, and Med7), Transport (Yet2, Sec1, Acb2, and Mth1), Nucleic Acid Metabolism (Tpp1, Rex3, and Lsm3), Cell Structure (Gim4, Atg26, and End3) and Stress Response (Ssb1). Three proteins (Mcpb, Ynr1, and Spg4) were not included in any of the categories, while two of them (Irc10 and Spac24c9.04) had unknown functions (Table 3).

**Table 3:** Categorization of proteins identified in *Candida albicans* 111R strain treated with EAF of *Eugenia uniflora* (1,000 μg/mL) after morphogenesis induction in fetal bovine serum after 3 h of incubation at 37°C, 200 rpm.

**Group of proteins**	**Gene**	**Biological process**
**ENERGY**		
Mitochondrial import inner membrane translocase subunit TIM16	PAM16	Mitochondrial biogenesis
F1F0-ATPase complex. F1 beta subunit	ATP2	ATP synthesis
Respiratory supercomplex factor 1. mitochondrial	RCF1	Mitochondrial biogenesis
Mitochondrial outer membrane protein	CAGL0G03245g	Mitochondrial biogenesis
Cytochrome c oxidase subunit 4. mitochondrial	COX4	ATP synthesis
Nucleoside diphosphate kinase	NDK1	ATP synthesis. Nucleotide metabolism
Protein PET191. mitochondrial	CAWG_02398	ATP synthesis
**PROTEIN METABOLISM**		
GTP-Binding Protein 1	GTP1	Protein syntesis
Peptidyl-prolyl cis-trans isomerase 9	CYP9	Protein syntesis
60S large subunit ribosomal protein L3	RPL3	Protein syntesis
Proteasome Subunit Alpha-2	PCA2	Protein Catalysis
54S ribosomal protein L4	MRPL4	Ribossomal structure
26S Proteasome regulatory subunit N6	RPN6	Protein Catalysis
ATP-dependent RNA helicase DBP10	DBP10	Ribossomal structure
Elongation factor 1-alpha 1	TEF1	Protein syntesis
ATP-dependent RNA helicase MRH4. mitochondrial	MRH4	Ribossomal structure
**GLUCOSE METABOLISM**		
Enolase 1	ENO1	Glycolytic pathway—Growth
Fructose-bisphosphate aldolase	FBA1	Glycolytic pathway—Growth
Mediator of RNA polymerase subunit 7	MED7	Glycolytic pathway promoter
**CELL DIVSION**		
Kinetochore protein Nuf-2	NUF2	Cell division
Cohesin	PDS5	Cell division
Spore Wall Protein 12	SWP12	Cell division
Meiotically up-regulated gene 97 protein	MUG97	Cell division
**TRANSPORT**		
Endoplasmic reticulum transmembrane protein 2	YET2	Protein transport
Syntaxin-binding protein 1	SEC1	Protein transport
Acyl-CoA-binding protein 2	ACB2	Protein transport
Protein MTH1	MTH1	Glucose transport. signal transduction
**NUCLEI ACID METABOLISM**		
Polynucleotide 3′-phosphatase	TPP1	DNA repair
RNA exonuclease 3	REX3	RNA processing
U6 snRNA-associated Sm-like protein LSm3	LSM3	RNA processing
**CELL STRUCTURE**		
Prefoldin subunit 2	GIM4	Protein folding
Sterol 3-beta-glucosyltransferase	ATG26	Membrane synthesis
Actin cytoskeleton-regulatory complex protein END3	END3	Endocytosis; Growth
**STRESS RESPONSE**		
Heat shock protein SSB1	SSB1	Protein biosynthesis. Stress response
**OTHER FUNCTION**		
Carboxypeptidase 2	MCPB	Extracellular metalloprotease
Nitrate reductase	YNR1	Nitrate assimilation
Stationary phase protein 4	SPG4	Stationary phase
**UNKNOWN FUNCTION**		
Ucharacterized protein	IRC10	Unknown function
Ucharacterized protein	SPAC24C9.04	Unknown function

From the total 39 proteins identified and classified, 23.1% were included in the “Protein Metabolism” category, generally associated with protein synthesis and structural formation of ribosomes. A total of 17.9% of identified proteins were included in the “Energy” category, and mitochondrial biogenesis and ATP synthesis were the main functions observed. The three proteins classified in “Glucose metabolism” group were related to glycolytic pathways. All proteins categorized in the “Transport” group were correlated with the maintenance of cell functions by promoting the transport of glucose and protein in the cytoplasm of *C. albicans* cells.

Proteins involved in the “Nucleic Acid Metabolism” were associated with biological functions of DNA repairing, RNA processing and protein folding. Membrane synthesis and endocytosis are functions related to the “Cell structure” group. Only a single protein (Ssb1) was included in the “Stress Response” group and its biological function is related to protein biosynthesis and stress response.

### Proteomics analysis of *Candida albicans* treated with the EAf of *Eugenia uniflora*

The pattern of *C. albicans* differentially expressed proteins in the presence of the EAF of *E. uniflora* is described in Table 4. Eight proteins were overexpressed in the presence of the EAF (Pam16, Med7, Atp2, Rcf1, Eno1, Ynr1, Spg4, and Ssb1), while 10 proteins showed reduced expression, as follows: Gtp1, Nuf2, Cyp9, Acb2, Mug97, Cox4, Yet2, Mth1, Ndk1, and Gim4.

**Table 4:** Proteins differentially expressed and corresponding biological process of *Candida albicans* 111R strain treated with EAF of *Eugenia uniflora* (1,000 μg/mL) after morphogenesis induction in fetal bovine serum after 3 h of incubation at 37°C, 200 rpm.

**Proteins**	**Gene**	**Biological process**
**INCREASE**		
Mitochondrial import inner membrane translocase subunit TIM16	PAM16	Mitochondrial biogenesis
Mediator of RNA polymerase subunit 7	MED7	Glycolytic pathway promoter
F1F0-ATPase complex. F1 beta subunit	ATP2	ATP synthesis
Respiratory supercomplex factor 1. mitochondrial	RCF1	Mitochondrial biogenesis
Enolase 1	ENO1	Glycolytic pathway—Growth
Nitrate reductase	YNR1	Nitrate assimilation
Stationary phase protein 4	SPG4	Stationary phase
Heat shock protein SSB1	SSB1	Protein biosynthesis. Stress response
**DECREASE**		
GTP-Binding Protein 1	GTP1	Protein syntesis
Kinetochore protein Nuf-2	NUF2	Cell division
Peptidyl-prolyl cis-trans isomerase 9	CYP9	Protein syntesis
Acyl-CoA-binding protein 2	ACB2	Protein transport
Meiotically up-regulated gene 97 protein	MUG97	Cell division
Cytochrome c oxidase subunit 4. mitochondrial	COX4	ATP synthesis
Endoplasmic reticulum transmembrane protein 2	YET2	Protein transport
Protein MTH1	MTH1	Glucose transport. signal transduction
Nucleoside diphosphate kinase	NDK1	ATP synthesis. Nucleotide metabolism
Prefoldin subunit 2	GIM4	Protein folding

Several biological processes were affected when the EAF of *E. uniflora* was added to *C. albicans* 111R NGY broth and morphogenesis was further induced. Proteins involved in generation of energy by ATP synthesis (Atp2), mitochondrial biogenesis (Pam16 and Rcf1) and glycolytic pathway (Med7 and Eno1) were overexpressed in the presence of the fraction. Heat Shock Protein Ssb1, involved in *C. albicans* stress response was also overexpressed in the presence of the EAF (Table 4).

Proteins involved in the structural organization of *C. albicans* were down regulated during morphogenesis after growth in the presence of the EAF. Cytoplasmic transport of proteins (Acb2) and cell division process (Nuf2 and Mug97) also showed decreased protein expression in the presence of the EAF of *E. uniflora*. Protein synthesis (Gtp1 and Cyp9) and folding (Gim4) were also negatively induced in the presence of the fraction (Table 4).

### Murine model of oral candidiasis

The effect of the EAF of *E. uniflora* in *C. albicans* hypha formation and pathogenicity was evaluated *in vivo* under three different conditions: Group I: *C. albicans* strain 111R grown in NGY broth the absence of the *E. uniflora* EAF. Group II *C. albicans* strain 111R grown in NGY broth the presence of *E. uniflora* EAF and Group III: *C. albicans* strain 111R grown in NGY broth in the absence of *E. uniflora* EAF, where the natural product was administered to the animal's tongue after infection.

**Figure 2:**
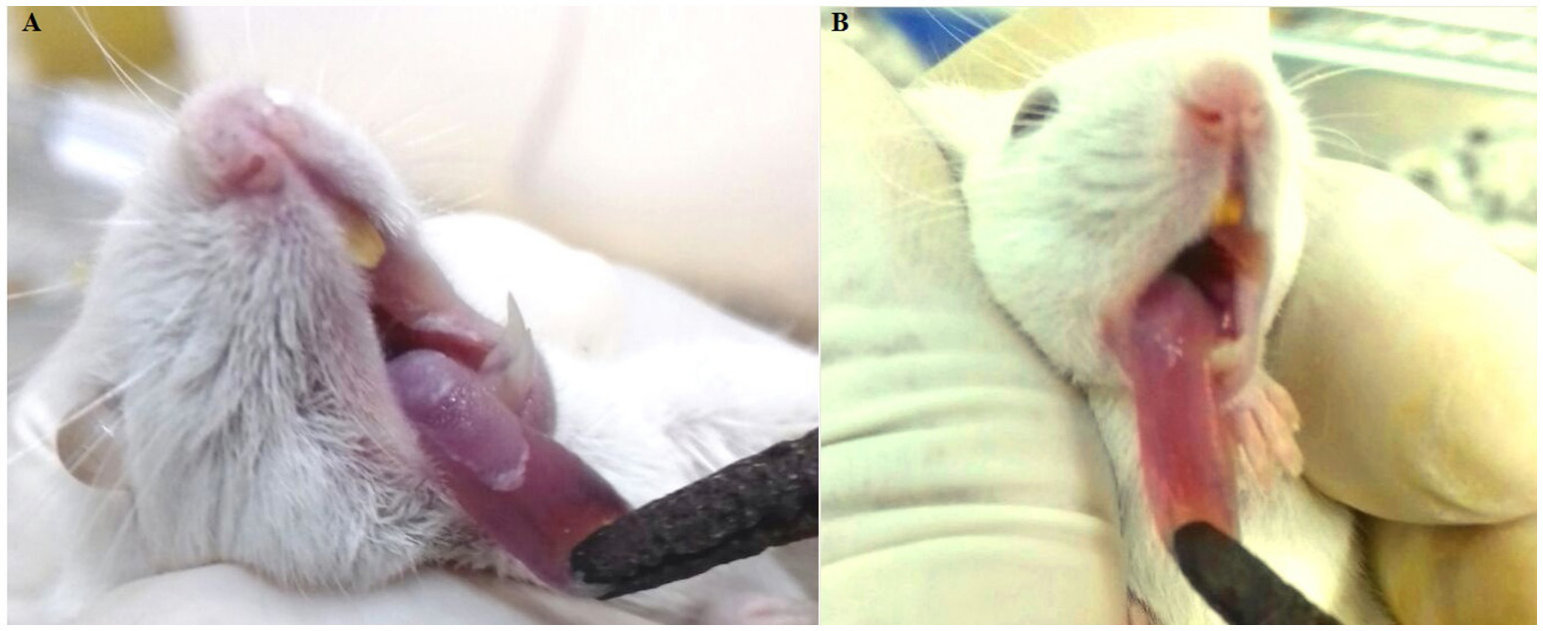
Murine model of oral candidiasis. **(A)** Macroscopic lesions of candidiasis on the tongue dorsum characterized by pseudomembranous white plaques in mice infected with *Candida albicans* untreated with *Eugenia uniflora* extract (Group I). **(B)** Aspect of lesion on the tongue dorsum in an animal infected with *Candida albicans* grown overnight in the presence of *E. uniflora* EAF (1,000 μg/mL;Group II), showing a remarkable reduction of confluent lesions on mucosal surface.

In the macroscopic analysis of the tongue dorsum of mice infected with *C. albicans* grown in the absence of the *E. uniflora* EAF, the presence of lesions with pseudomembranous white plaques corresponding to establishment of oral candidiasis was observed (Groups I and III; Figure 2A) while the group of mice infected in the oral cavity with 111R strain grown overnight in the presence of the EAF of *E. uniflora* was macroscopically less affected with the infection and pseudomembranous white plaque in the dorsum of tongue was not observed (Group II; Figure 2B). Two days after the infection, the animals from group III were treated by administering the fraction on the surface of the tongue, reducing the progression of the formation of white pseudomembranous confluent plaques when compared with group I, which did not received treatment.

**Figure 3:**
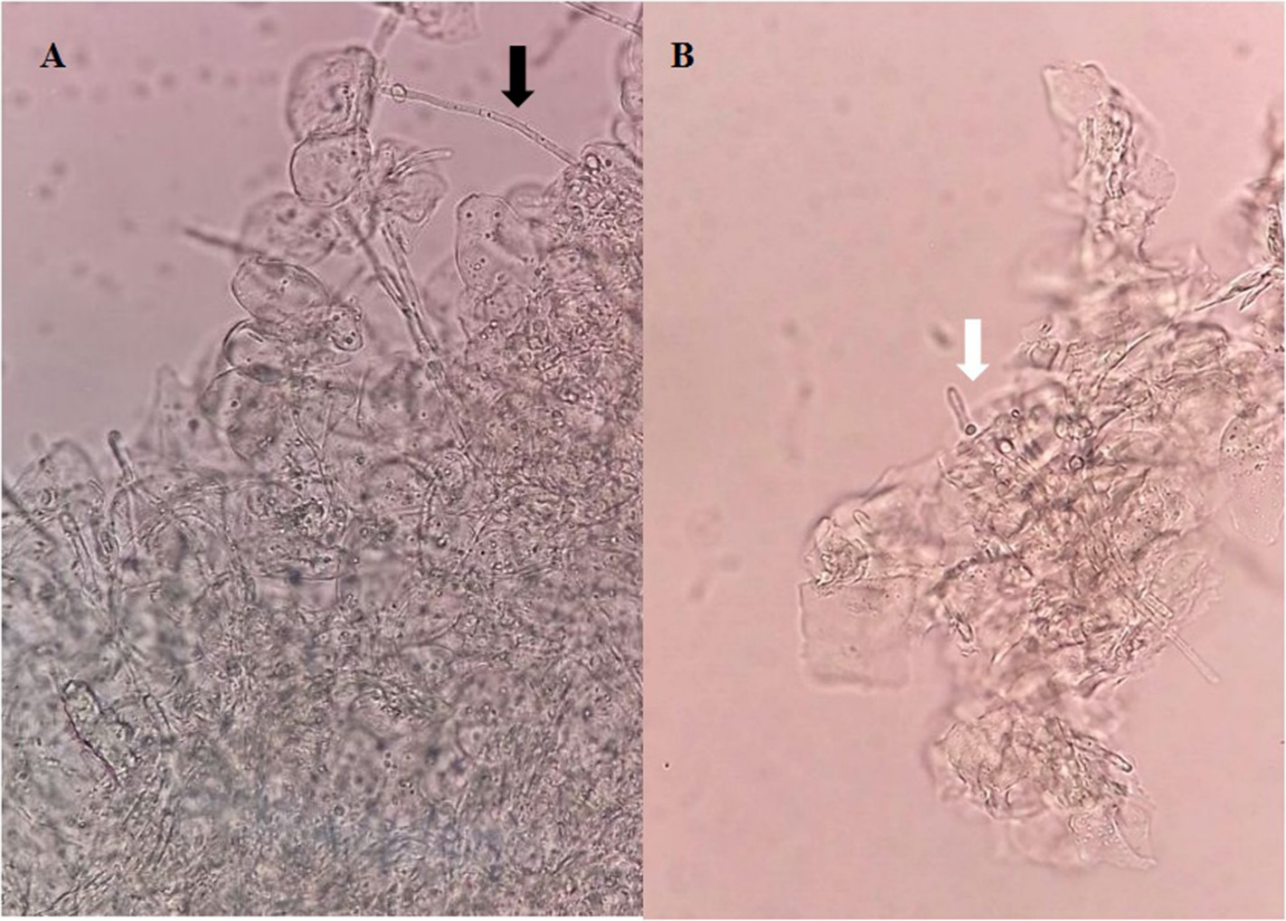
Direct examination of a biological sample collected from the oral cavity of mice infected with *Candida albicans* 111R untreated with *Eugenia uniflora* extract—Group I **(A)**, and **(B)** *Candida albicans* 111R strain previously grown in the presence of *Eugenia uniflora* EAF (1,000 μg/mL)—Group II. Black arrow indicates a long true hypha characteristic of *Candida albicans* 111R strain. White arrow shows a reduction of hyphal size. Asterisk indicates oral epithelial cells, in higher proportion in Group I rather than Group II. Optical microscopy, 400× of magnification.

A sterile swab was rubbed in the oral cavity of animals and introduced into a tube containing 1 ml of 0.9% saline to perform direct examination and culture on SDA to analyze the fungal burden by CFU/ml counts. In the microscopical analysis of the sample collected from the mice belonging to Group I, we observed the presence of cellular clumps of long hyaline and septate hyphae (Figure 3A). The direct examination of samples obtained from mice belonging to Groups II and III showed visually shortest pseudo-hyphae and true-hyphae (Figure 3B). This difference was more pronounced for Group II samples.

**Figure 4:**
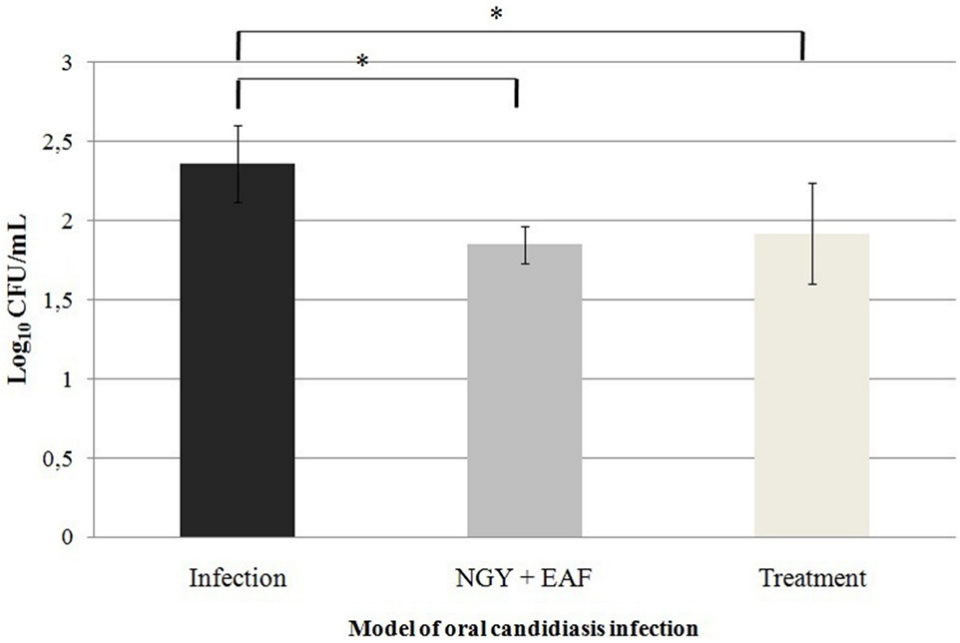
Mean values and standard deviations of the number of CFU/mL of *Candida albicans* 111R recovered from lesions on the tongue dorsum of mouse in murine model of oral candidiasis. Samples were seeded on SDA, incubated at 37°C, 48h. ^*^Significant difference between the number of CFU/mL recovered from the animals of the different groups of infection, *P* < 0.05.

**Figure 5:**
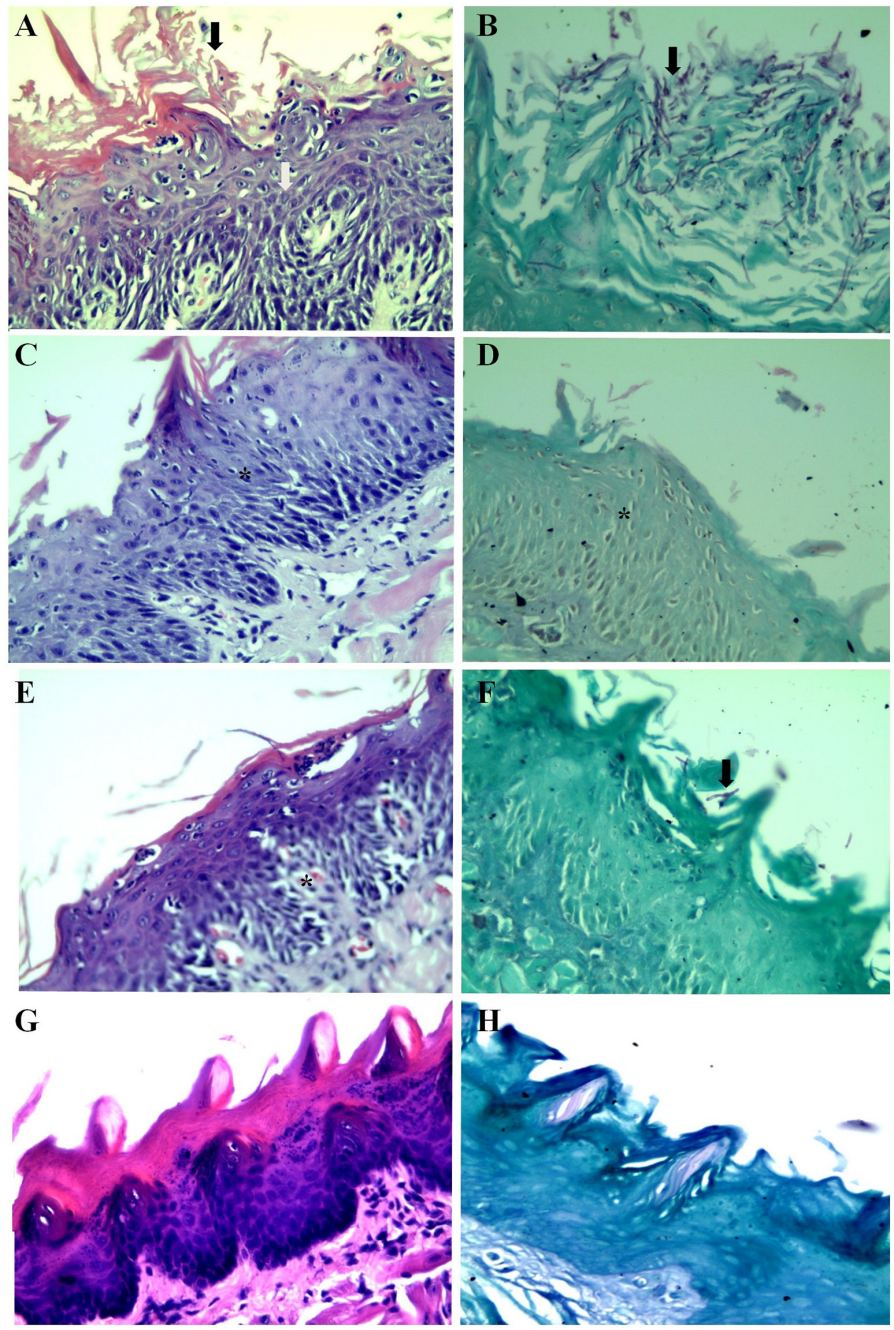
Histopathological analysis of the dorsum of the tongue of mice. **(A)** Group I, HE, Stratified squamous mucosa with acanthosis, spongiosis, severe neutrophilic exocytosis (white arrow) and erosion of the epithelium (black arrow). **(B)** Group I, PAS, Stratified squamous mucosa exhibiting numerous corneal layer and pseudohyphae and true hyphae (filamentous structures) of fungi (black arrow). **(C)** Group II, HE, Stratified squamous mucosa with acanthosis, spongiosis and mild exocytosis (asterisk). **(D)** Group II, PAS, Stratified squamous mucosa without evidence of fungi (asterisk). **(E)** Group III, HE, Stratified squamous mucosa with mild acanthosis, spongiosis and mild exocytosis (asterisk). **(F)** Group III, PAS, Stratified squamous mucosa with rare short pseudohyphae black arrow) **(G,H)** Normal squamous mucosa without evidence of lesions and fungi.

Oral cavity samples were seeded on the surface of SDA, incubated at 37°C to perform CFU counting. The number of CFU/mL is described in the Figure 4. A reduction in the number of CFU from 2.36 Log_10_ CFU/ml to 1.85 Log_10_ (Groups I and II, respectively) and from 2.36 Log_10_ CFU/ml to 1.92 Log_10_ (Groups I and III, respectively) was observed. The histopathological analysis of the mice tongue infected with *C. albicans* was performed. The mucosa damage and fungal presence were evaluated with HE and PAS staining. The presence and absence of ulceration, erosion, inflammatory response and type of inflammatory cells were analyzed (Figures 5A–H). The animals belonging to Group I showed both more extensive tissue damage and fungal presence, specifically filamentous forms (long pseudo-hyphae and true hyphae), while the animals belonging to Groups II and III had less tissue damage and fungal presence (Figures 5A–H).

### Cell viability assay

**Figure 6:**
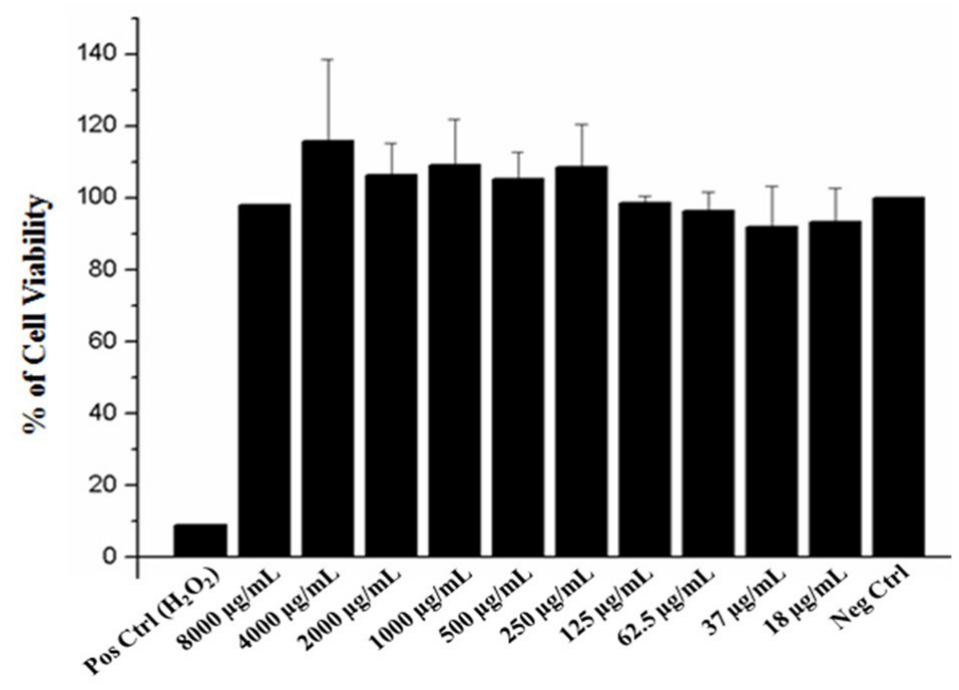
Effect of the EAF of *Eugenia uniflora* on A549 cell line viability determined by MTT assay. Pos Crtl (control); tested concentrations of the extract (8,000–18 μg/). The samples were analyzed in three independent experiments performed in duplicate.

To ensure the safety and evaluate the cytotoxic effect of the EAFof *E. uniflora* in human cells, a MTT assay was performed using A549 cells, in concentrations ranging from 8,000 to 18 μg/mL. The extract was not toxic against the cells tested, which showed high viability over than 80% of cells even when the fraction concentration was 8-folds higher than the concentration used to perform all the experiments of the present study (Figure 6).

## Discussion

In order to investigate the effect of the *Eugenia uniflora* EAF in protein profile of *C. albicans* and in a murine model of oral candidiasis, we isolated various *C. albicans* strains from renal transplant recipients. A phagocytosis assay was performed and the highly hypha-forming strain 111R presented the lowest number of phagocytized *C. albicans* cells (11 phagocytized cells in 100 PMNs), while the other isolates were highly phagocytized. This fact reflects the importance of filamentation against the action of phagocytic cells, corroborating the idea that the emission of a long germ tube is a crucial factor to escape the immune system.

Fradin et al. (2005) described that when incubated in the presence of PMNs (in whole blood and plasma), 95.6% of *C. albicans* cells are still in the form of blastoconidia, 38% of these cells are phagocytized, while 57.5% are bound to PMNs after 30 min incubation. Based on these data, the strain 111R was not effectively phagocytized due to the fact that when previously grown in NGY broth, this strain already presents a large number of hyphae formed, even at a temperature that does not stimulate filamentation. Thus, when incubated in the presence of PMNs, cells were not efficiently phagocytized.

On the other hand, for most of the strains, a significant reduction occurred in the number of *Candida* cells phagocytized by the PMNs, if previously grown in the presence of the fraction. This finding reinforces our hypothesis that the *E. uniflora* EAF is acting on the *C. albicans* cell wall of due to the fact cells were previously grown in the presence of the referred natural product. Therefore, some probable accumulation of components of the EAF in the cell wall may impair PMNs recognition and interaction with β- glucans, mannan and mannose receptors, which are part of the constitution of the cellular wall, and recognized by pattern recognition receptors (PRPs) found on the surface of defense cells (Gow et al., 2012; Erwig and Gow, 2016).

Our unexpected results of PMNs phagocytosis reduction after treating *Candida* cells with *E. uniflora* does not invalidate our natural product as a possible antifungal drug in the future, because others have found the same effect with amphotericin B and this is still a very broad spectrum and highly effective antifungal drug used in the clinical practice (Pallister et al., 1992).

Our proteomics analysis revealed that most of the proteins expressed were related with Protein Metabolism (23.1%) and Energy (17.9%). This fact is possibly related with the morphogenesis assay, because protein synthesis is highly necessary to the process of transition from yeast to hyphae, as well as ATP synthesis to provide energy for the construction and elongation of the cell wall of *C. albicans* is another crucial step (Dudek et al., 2013).

In the presence of the EAF of *E. uniflora*, proteomics analysis showed that eight proteins were overexpressed, including Pam16. The transport of preproteins into the mitochondrial matrix depends of the association of Presequence Translocase of the Inner Membrane (TIM23) and Presequence Translocase-Associated Motor (PAM) which is a complex of proteins that form an aqueous channel for the transport of presequence-carrying preproteins into the mitochondrial matrix, and the PAM complex includes the Pam16 protein (Li et al., 2004). In this complex, the Pam16 function is to form a stable complex with Pam18 and antagonize this protein in its function of promoting the regulation of ATPase activity of mtHSP70 protein in the mitochondrial matrix (Dudek et al., 2013). The overexpression of Pam16 suggests a reduction in the metabolism of *C. albicans* cells in the presence of the fraction because it is a negative key regulator acting directly in Pam18 reducing ATPase function.

F1F0-ATPase complex beta subunit (Atp2) is a cell surface protein (Gil-Bona et al., 2015) associated with ATP synthase complex proteins, participating in the process of cell respiration and energy. In a study of proteome profiling of *C. albicans* (Monteoliva et al., 2011), a reduced expression of Atp2 in the differentially detected proteins related with the respiration process in the yeast-to-hyphae transition was described. Ebanks et al. (2006) investigated *C. albicans* yeast and hyphal cell wall and associated proteins by proteomics, and observed that Atp2 and Eno1 were down regulated during the hyphal stage, when compared with yeast forms. This fact may explain the reason why both proteins are overexpressed in our cells treated with the EAF that cause a reduction in filamentation.

Enolase 1 (Eno1) is an important protein of *C. albicans* involved in several biological functions (Silva et al., 2014). The main function of Eno1 is in the glycolytic pathway, where it catalyzes the conversion of 2-phospho-D-glycerate to phosphoenolpyruvate, which is critical in both glycolysis and gluconeogenesis (Ko et al., 2013). Enolase 1 also participates in several biological functions, as a heat shock protein involved in thermotolerance and growth (Silva et al., 2014), suggesting that the high expression of Eno1 in our treated cells could be a compensatory mechanism of *C. albicans* to survive to the adverse condition of the *E. uniflora* EAF associated with the incubation at 37°C.

Several proteins were down-regulated in the presence of the *E. uniflora* EAF and the main biological processes associated with these proteins were protein synthesis and transport (Gtp1, Cyp9, Acb2, Yet2), ATP synthesis (Rcf1, Ndk1 and Cox4), Cell division (Nuf2, Mug97), Glucose transport (MTH1) and Protein folding (Gim4). The biological processes affected by the reduction of these proteins could be related to the formation of fragile and damaged cells, with reduced metabolism and difficult growth.

The Kinetochore protein Nuf2, which is associated with cell division, was down-regulated in our treated cells. Kinetochore is a protein complex machine localized on the centromere of each chromosome. The primary function of kinetochore is to lead to the attachment of the chromosome to the dynamic plus ends of spindle microtubules. This is an important step in the segregation of chromosomes (Roy et al., 2013). It is also related with the formation of heterochromatin and maintenance of cohesion between sister chromatids until anaphase onset and is also involved in the recruitment of the spindle assembly checkpoint machinery to prevent cell cycle progression if an error persists (Roy et al., 2013). In fact, if there are failures on the kinetochore machinery either by repeating a subunit proteins or lacking ones, the interaction of kinetochore-microtubules in meiosis organization may result in aneuploidy due the unequal distribution of chromosomes (Joglekar et al., 2008). Therefore, our cells may be unable to properly complete cell cycle, compromising all cellular metabolisms.

Cytochrome C oxidase subunit 4 (Cox4) belongs to the electron transport chain in *C. albicans* (Schmidt et al., 2010; Mick et al., 2011). Cox4 expression was reduced in our treated cells. These results are in agreement with Gil-Bona et al. (2015), which found that Cox4 was more found in hyphae rather than budding cells, corroborating with the fact that filamentation was reduced in our cells treated with the natural product.

Several proteins are involved in the organization of cytoskeleton during *C. albicans* morphogenesis, an essential step that contributes to yeast-to-hyphae transition (Millan-Zambrano and Chavez, 2014). Prefoldin was down regulated in our treated cells. This is a multi-subunit complex protein containing six polypeptides with molecular mass ranging from 14 to 23 kDa (two α-subunits and four β-subunits) (Simons et al., 2004; Millan-Zambrano and Chavez, 2014). Prefoldin is responsible to the important function of bind to native and unfolded tubulin and actin and delivery to Chaperonin-containing T-complex polypeptide-1 (CCT), the protein responsible to the folding of these proteins and its assembly into high-order protein structures, such as microtubules and actin filaments, which are essential to the cytoskeleton organization (Millan-Zambrano and Chavez, 2014).

In order to investigate the *in vivo* effect of the EAF of *E. uniflora*, a murine model of oral candidiasis was established with the 111R strain pre-cultivated in the presence and absence of the natural product. When the infection was established with *C. albicans* untreated cells (control), the presence of a confluent pseudomembranous white plaque on the tongue surface, typical of the oral candidiasis lesion was observed (Millsop and Fazel, 2016). These lesions were significantly reduced when *Candida* cells were pre-cultivated in the presence of the *E. uniflora* EAF, because the animals presented slightly white spots dispersed on the tongue surface and absence of macroscopic lesion. The reduction of lesions agreed with the findings at the direct examination. *C. albicans* cells obtained from Group I animals presented aggregates of long fungal filaments associated with large cell desquamation, observed by the presence of numerous epithelial cells in direct examination. The presence of visually shorter hyphae and the reduction of epithelial desquamation were observed in previously treated cells as well as when the infection was established and then the EAF was administered on the tongue surface. The macroscopic reduction of lesion aspect and reduction of presence of hyphae and viable cells was confirmed with the Colony Forming Units (CFU) assay. In the presence of the *E. uniflora* extract, there was a significant reduction in *C. albicans* CFU growth.

The reduction in CFU counting observed in our study reflects the action of the EAF of *E. uniflora* in *C. albicans*, causing damage to the cells and death, which was reflected in the reduction of CFU in both conditions tested and may be directly linked to initial impairment to form hyphae during the experiments *in vivo*.

In order to evaluate the essential oil of *Melaleuca alternifolia* for the treatment of oral candidiasis induced in an immunosuppressed mouse model, de Campos Rasteiro et al. (2014) induced oral candidiasis in the same condition of the present study and after the establishment of infection, the essential oil was administered on the tongue surface. *C. albicans* cells were recovered from the tongue with a sterile swab and it was observed a significant reduction of CFU counting when the essential oil was administered, indicating the lethal effect of the *Melaleuca alternifolia* on infection by *C. albicans*. However, its main component terpinen-4-ol was less efficient than fluconazole to treat mice with experimentally induced oral candidiasis, where no yeast colonies were recovered and tongue lesions were not detected (Ninomiya et al., 2012).

The extract of North American ginseng (*Panax quinquefolius* L.) in *C. albicans* disseminated infection was evaluated by administering the extract in the animal's drinking water in a 1% solution provided to mice for *ad libitum* ingestion 48 h before experimental infection. Heart, Brain and both kidneys were recovered for *C. albicans* culture. The efficacy of the *Panax quinquefolius* extract was observed by the higher survival of animals and reduction im CFU counting, mainly in the kidneys (Trammell et al., 2012). Therefore, our results strongly suggest that *E. uniflora* impairs proper hypha formation in *C. albicans* and this phenomenon may reflect directly in pathogenesis which may be observed in the decrease of CFU counting in yeast treated cells.

The reduction of hypha formation by *C. albicans* 111R observed *in vitro* (Silva-Rocha et al., 2015) was confirmed *in vivo* in the present study. Our histopathological findings corroborate with the macroscopical lesion analysis, because in the animals where the infection was established with *C. albicans* cells pre-treated with the EAF of *E. uniflora*, the reduction of filamentation was also observed in both HE and PAS staining examination and consequently, tissue damage was reduced. The same findings were observed in the study of de Campos Rasteiro et al. (2014) using the essential oil of *M. alternifolia* in oral candidiasis. In this study, the essential oil was also capable to considerably reducing the invasion of *C. albicans* on the tongue tissue and it was evidenced by the observation of fewer lesions in the animals when compared with the control group.

In order to evaluate cell viability and ensure the safety of use of *E. uniflora* the EAF in human cells, a MTT assay was performed using an epithelial lung cell line A549 and the cell survival rate was very high, with more than 90% of the viable cells in the concentration of 8 mg/mL and this fact may reflect safety of the EAF that may be used in the future as an antifungal compound.

Several studies are performed to analyze the safety of natural products extracts with antimicrobial activities by MTT. Recently, de Oliveira et al. (2017) evaluated the biological activities of *Rosmarinus officinalis* L. (rosemary) extract (25, 50, and 100 mg/mL) in *C. albicans, Staphylococcus aureus, Enterococcus faecalis, Streptococcus mutans* and *Pseudomonas aeruginosa* cells. It was observed that it was necessary a concentration over than 50 mg/mL of the extract to reduce 40% of the cell viability using human gingival fibroblasts (FMM-1), human breast carcinoma cells (MCF-7), and cervical carcinoma cells (HeLa).

Madeira et al. (2016) evaluated the *Cymbopogon citratus* (i.e., lemongrass) extract in *C. albicans* cells and cytotoxicity was analyzed by MTT using peripheral blood mononuclear cells (PBMCs) collected from healthy human volunteers. It was observed that a reduction of 40% of viability occurred when the cells were treated with 10xMIC. In the same way, other natural products have been considered of low toxicity, our findings have shown that the EAF of *E. uniflora* may be safe for the application in the prevention or treatment of *C. albicans* superficial infection.

## Conclusions

The emergence of new therapeutic alternatives for the treatment of oral candidiasis is necessary and the use of natural products has increased recently. The findings demonstrated in the present study suggest that the EAF of *E. uniflora* acts on the cell wall of *C. albicans*, interacting directly with important proteins involved in the structural organization of *C. albicans* morphology and essentials pathways of metabolism, which can be reflected in the damage to its structure, reducing filamentation, which made cells defective and unviable. This fact is reflected with the significant reduction of lesions observed macroscopically on animal's tongue, as well as in the reduction of invasion and tissue damage, observed with histopathologycal analysis. In addition, in human cells, even at concentrations eight times above the concentration used in the present study, the *E. uniflora* EAF did not show cellular cytotoxicity, which probably makes it safe for administration in human cells. Thus, the EAF of *E. uniflora* may be a potential new therapeutic agent for the treatment of superficial candidiasis in the future.

## Ethics statement

This study was carried out in accordance with the recommendations of Law number 11,794, of October 8, 2008, of Decree number 6,899, of July 15, 2009 and with the regulations issued by the National Council for Control of Animal Experimentation (CONCEA) of the ETHICS COMMITTEE IN THE USE OF ANIMALS (CEUA), Federal University of Rio Grande do Norte. The protocol was approved under the number 034/2015.

## Author contributions

WS performed the phenotypic tests, proteomics analysis and murine model experiments, analyzed data and prepared the manuscript. MdA performed animal experiments, MF and LS obtained and provided the *E. uniflora* EAF, JdS performed the MTT assay and supported MALDI-TOF analysis. TS and EM isolated and identified *C. albicans* strains, KR performed histopathological analysis, AU supported with protein extraction purification and bidimensional gel electrophoresis, MM and AF supported MALDI-TOF analysis, GC designed all tests and prepared the manuscript. All the authors approved the final manuscript.

### Conflict of interest statement

The authors declare that the research was conducted in the absence of any commercial or financial relationships that could be construed as a potential conflict of interest.
